# Americocentrism and Art of the Caribbean: Contours of a Time–Space Logic

**DOI:** 10.1017/S0021875813000145

**Published:** 2013-05

**Authors:** LEON WAINWRIGHT

## Abstract

Art of the transnational Caribbean has come to be positioned by an understanding of the African diaspora that is oriented to an American “centre,” a situation to be explored for what it reveals about the hegemonic status of the United States in the discipline of contemporary art history. The predominant uses of the diaspora concept both in art-historical narratives and in curatorial spaces are those that connect to United States-based realities, with little pertinence to a strictly transnational theorization. This has implications for how modern art and contemporary art are thought about in relation to the Caribbean and its diaspora, in a way that this article demonstrates with attention to a number of artists at multiple sites, in Trinidad, Guyana, Britain and America.

In the field of modern and contemporary art, understanding of the transnational Caribbean has come to be oriented to an American “centre.” It is not simply that the US Library of Congress has sometimes classified art of the Caribbean as “African American,” but that the region has been positioned by conceptualizations of the African diaspora that correspond with a locus of historical experiences that belong properly to the United States. The situation is worth exploring further for what it discloses about the hegemonic role of the United States in defining art in both the Caribbean and its diaspora in Britain.

There have been numerous attempts to assemble a history of art for the African diaspora through art exhibitions and art-historical studies that seek to connect artists from across a wide geography which is emphatically transatlantic and transnational. Their aim has been to mobilize ideas of the African diaspora and to assert the historical value of its art – thereby seeking to combat the traditional exclusion of diaspora communities from public and institutional memory. However, this intention seems to be in conflict with what may be described as the Americocentrism that has ensued from such representations in their treatment of art and artists associated with the Caribbean. It is a conflict so deep as to suggest that the diaspora concept itself, which once promised new departures for imagining community beyond the nation, has rather lost its trans- or internationalist emphasis. It may instead have become susceptible to political and social priorities located in a certain national setting – those focussed on the United States – which are normative in the treatment of diaspora culture in other locations across the Atlantic region.

As I explain in this essay, exploring the background to these circumstances means drawing attention to the broader history of America's standing in a wider Atlantic history of modern and contemporary art. In this critical geography of art, considerations of power have to be made in relation to time as much as to space, and through the dynamic intersections of them both can be uncovered a “logic” for art history. In what follows, I suggest how the movements and lines of connection exemplified by three artists with Caribbean backgrounds may provide a basis for discussing a complex field that extends across frontiers, intersecting multiple sites of the Caribbean – namely Trinidad and Guyana – as well as the United States and Britain.

## ART HISTORY'S TIME–SPACE LOGIC

To help make sense of these relationships it is worth bringing out two distinctions. The first, and more easily recognized of the two, belongs to a group of conceptualizations about time and space which attempts to explain for differences of cultural experience. It is a rationalisation that assumes certain places to provide the conditions for creativity and innovation: perceived “centres” where “new” and “original” cultural products – among them works of art – are most likely to be found. These are the prime conditions for supporting, even for driving, cultural development, and in the history of artistic modernism they may be contrasted with the greater number of other places which are thought to somehow lag behind, or cling to the past. Seen in this way, there is far greater status for those centres of change which lead or precipitate innovation across a wider geography that is yet to “catch up.” They are places where change is first found to be in evidence, as well as having the effect of bringing about change at the margins, so that these peripheral locations may effectively be brought into being or granted status through their centre's influence.

Such a relationship between centres and margins is in part what David Summers has identified through his attention to the process of “the appropriation of the centre,” when the creation of “the point where the world is defined, with its values of collective generation, and the combination of notional ‘cosmic’ order and vital centrality provides the political order.”^1^ This scheme – of “leading” centres and “backward” peripheries, in which political meaning is bound up with the cultural values of “generation” – was normalized in imperial visions of global social evolution, yet could be overlain just as readily on a less expanded geography such as the domestic territory of a nation, with its metropole and provinces. Among the pillars of modern techno-bureaucratic development were representations of political power which animated the geography of empires as much as the complex spatiality of modern nationhood: they were foundational to arguments about the smooth running of political units, and the legitimacy of the power of the “centre.” Indeed Marx would write, as poignantly repeated in Said's epithet to his study *Orientalism*, “They cannot represent themselves; they must be represented.”^2^.

It is worth assessing how art history, an epistemological project that encompasses the intellectual work of museums, appears in the light of this first distinction. Of those who “cannot represent themselves” at centres of political power there is an attendant supposition that – whether subjects of colony, empire or nation – “they” would *eventually* share the means of representation. Such spaces between “us and them” are consequently marked out in temporal terms. I am interested in how this has happened in the assembling of histories of art. As I will suggest, its operational structure is similar to what self-reflexive anthropologists such as Johannes Fabian, as well as many postcolonial theorists of the 1980s, had noted about disciplinary practices of differencing:

the temporal discourse of anthropology as it was formed decisively under the paradigm of evolutionism rested on a conception of Time that was not only secularized and naturalized but also thoroughly spatialized. Ever since, I shall argue, anthropology's efforts to construct relations with its Other by means of temporal devices implied affirmation of difference as distance.^3^

If anthropology shares anything with art history it is these outlines of a “time–space logic,” one for which familiar narratives of art (with their vocabularies of innovation, creativity, change and continuity, and of the transmission of “style” or “tradition” and so on) have been mapped geographically, and most typically in relation to temporally “leading” centres. As unequivocally advanced, such centres not only render the peripheries “backward,” or at least belated, they also represent a stage of development whose status is gauged by an ability to direct the path of change for the peripheries, and whose worth can be measured in the spatial extent of their influence.

Alongside this, a second distinction may be arranged, as well as some telling instances when this distinction has been set against the first. This other scheme of time and space is distinctive, and may be thought an alternative, since it breaks through – but, as I will suggest, does not always entirely break with or break away from – those imperial, national and North Atlantic-focussed understandings of social life and historical change that have underpinned art history's conventional geography. Such a distinction was made vivid in the writings of George Kubler, such as his book *The Shape of Time* of 1962, where on the matter that I have broached of the spatio-temporal aspects of cultural value he observes, “The genuine precursor usually appears upon the scene of a provincial civilization, where people have long been the recipients rather than the originators of new behaviour.”^4^ Kubler's use of the term “provincial” summons up the military origins of the term, as conquered territory (from the Latin *vincere*): a spatial metaphor that is equally to do with strategy as with geography, and whose genealogy is instructive for unpacking tensions and points of resistance.

I ought to put this for a moment into the context of early 1960s America. It is helpful to recall that Kubler's interest in the “precursor” and “new behaviour” summarized a train of avant-garde thinking among those artists and art historians who felt unable to accept the assumption that there is a single “development” in which all art may or should be placed. As Kubler put it, “the history of art is like a vast mining enterprise, with innumerable shafts, most of them closed down long ago.”^5^ This is, in essence, the counter to my first “centrist” distinction. It is one of the reasons why, throughout the 1960s in the United States, several artists drew direct inspiration from Kubler, such as Robert Morris, Ad Reinhardt and, most notably, Robert Smithson – as first suggested by his 1964 working notes for his neon sculpture *Eliminator* and subsequent writings.^6^ The geographical or geopolitical in Kubler's writings has held much subsequent value. At the very least, it signals the recognition of “many histories of art [which are] made up of open numbers of more or less local interactions,” as David Summers has described Kubler's contribution;^7^ in other words, the recognition of pluralism and discontinuity (traced through multiple sequences and series), in contrast to a persistent, linear and successive story.

**Figure 1.**
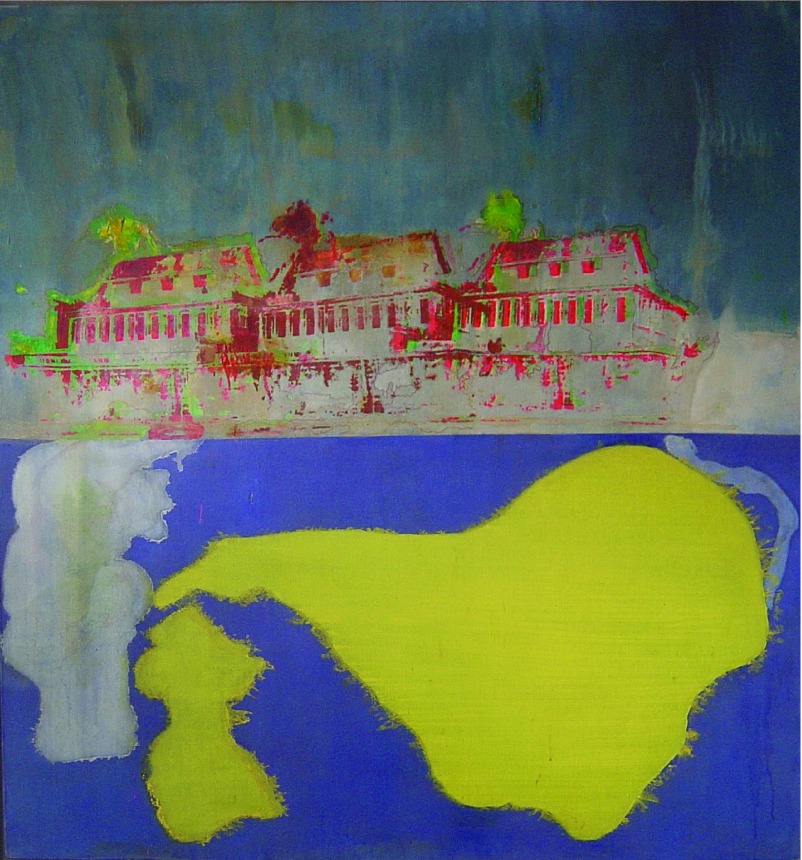
Frank Bowling, *Mother's House* (1967), acrylic on canvas, 157·5 × 167·6 cm. Courtesy of the artist.

In trying to understand the impact of these two distinctions, and why they have persisted for the ordering of art-historical representation, it is worth showing how the United States has served as a locus for some transatlantic relationships in the postwar period, and its bearing on the Caribbean and its diaspora in Britain. Admittedly, there is nothing like a traceable line of influence between Kubler and British art of the 1960s, such as there is in the American avant-garde (and the desire to trace one out would demonstrate the sort of weak historicism that Kubler abjured). But when we come to assess the role in British art of that period by an artist of the Caribbean – the British Guiana-born painter Frank Bowling (b. 1936; see Figure 1) – we can see the appeal, both to his British contemporaries and to their commentators, of Kubler's insistence on how “originality” may take shape at “the provinces.”

Frank Bowling studied at the Royal College of Art, among the soon-to-be-canonized artists associated with pop art (namely David Hockney, Derek Boshier and R. B. Kitaj). But before asking why he was not included in that canon and has largely been overlooked in accounts of British art, we would do well to remember how the United States held a growing presence in the ways that artists and their art were presented within Britain's national art story.

During the early 1950s, from within the United Kingdom's state of economic austerity, the graphic art and advertising that arose from the consumerism of the United States were viewed with envy and desire, and handled like cult objects heralding a seductive future. New York was clearly “ahead,” when the Independent Group, a forerunner to British pop art, saw London as a locus for dated design, of art “in arrears.”^8^ Indeed, so fascinated were British artists with American popular culture that often commentators have needed to remind gallery-goers of the sequence in which interest in pop grew. Brauer *et al.* write of pop's transatlantic historiography,

Pop Art has often been considered an essentially American phenomenon, but in fact British artists and theorists in the 1950s were the first to debate and formulate Pop's main tenets … Both the British and American artists who were eventually grouped under the rubric of Pop Art looked toward the United States as the primary source of this subject matter.^9^

At the same time, admiration for American popular culture would be mixed with disdain for its global spread, echoed by Peter Fuller's report of Britain's “bewildered recognition of the reality of a decadence, characterised by subservience to empty American fashions, and an understandable dwindling of the public for the newest art.”^10^

Like Fuller's, much of the writing about pop art in Britain has commiserated with critics from the period who felt that Britain's global position was weakening in the arts, as compared to an irrefutable American leadership. The pop artist Len Deighton summarized this view, writing,

although the Royal College of Art student body – with its ex-service students keen to earn a living – had no time to spare on politics, anti-Americanism was well-established there. Tweedy gentlemen-artists, who resented the thought of American bombers flying over “Constable country” found common cause with leftist students who loudly proclaimed that American design meant only Coca-Cola billboards and large cars with too much chromium.^11^

There was evidently a closing of ranks between these generations in order to critique high art's elitism. Quite apart from the obvious paradox of a left-wing embrace of American popular culture, with its material trappings of an advancing capitalism, this signalled a state of confusion about Britain's relation to the United States in the cultural field.

Maintaining a vocabulary of “the leading” and “the led,” there were variously expressed anxieties about Britain's sometime leadership having become superseded by that of America. A good example of the reassurances offered about such a development is the discussion generated by the exhibition Art in 1946 and After, when M. H. Middleton, writing in *The Spectator*, predicted that although Britain might be shedding “the political commitments of a great world power,” the country seemed destined “to hold a position of leadership we have never previously known as the artistic centre of the world.”^12^ This would become a running thread in the presentation and reception of art in the later twentieth century. In 1976, R. B. Kitaj made a return to this theme in the catalogue to the landmark exhibition The Human Clay: “The bottom line is that there are artistic personalities in this small island more unique and strong and I think numerous than anywhere in the world outside America's jolting artistic vigour.”^13^ In disagreement, an exhibition of 1987 at the Royal Academy attempted “to challenge the stereotyped views of the shortcomings of twentieth-century British Art, which for too long has been bedevilled by a reputation for politeness, provincialism and even timidity.”^14^ Trying to restore its reputation came with a chauvinistic and anti-American response to the exhibition from Peter Fuller. He concluded that British art of the twentieth century is best seen in “those resistances to, and refusals of, the worst aspects of modernity, and Americanism, which had previously ensured the distinctive, conservationist qualities of the British tradition.”^15^

A key moment in this history was the art of pop, and it saw writers absorbed in accounting for these two national locations – Britain and the United States. If typical accounts described them as enjoying a lively economy of aesthetic and social relations, there was also an emphasis on those countries' temporal as much as geographical distance. Several influential discussions of pop vividly connect Britain to the United States;^16^ others suggest areas of perceived equivalence with America, as well as crucial discontinuities.^17^ Their shared concern with national units is significant of how reflections on pop art in Britain even at its outset were dominated by an emphasis on place and locality. To borrow Lawrence Alloway's phrase to describe Paolozzi, the challenge for Britain was to defend its seeming inviolability against being “bombarded by mass media imagery” (the international visual culture considered to have emerged from an American epicentre), and to maintain its historical “lead.”^18^

This was evidently a moment in Britain's cultural history when an experience of provincialism and an attendant belatedness became the basis for advancing Britain's national interests within an Atlantic history of art. In other words, while seeking stances of opposition to America, and alternative paths for the development of modern art in the British art academy, the fact of Britain's art scene being overshadowed, even displaced or rendered “backward,” by North America became implicit in how the Britishness of its art was invented and celebrated.

Certainly, in only one respect could Britain never find comfort in presenting itself as “out of date,” and that was with regard to the decolonizing territories of its empire. This was why artists of the Caribbean who participated intimately in the art “scene” of London, such as Frank Bowling, were left out of the establishment exercise to reposition Britain vis-à-vis the United States. Such a process suggests that embracing the second distinction I have given – taking up the positional politics of outsiderness, as did the British mainstream relative to America – may at the same time repeat the first distinction, by enacting exclusions upon artists of the Caribbean. This was a fraught triangle of positions, which saw the United States, Britain and the Caribbean immersed in a nexus of centre–periphery relations.

The growing Caribbean diaspora in the UK was a community that meditated on whether cultural practices could overcome such histories. Intellectuals from parts of the Caribbean that were undergoing decolonization would focus on the significance for political independence of their own literature, painting and sculpture.^19^ The orientations toward the United States in the field of modern art were implicit in the challenges that Caribbean creativity would face, not least when Caribbean subjects moved to Britain. For discourses on art during the late 1950s and early 1960s, a sense of provincialism and its temporal connotation of belatedness presided over transatlantic connections to America, which for Britain made the matter of its shrinking empire all the more central. This held lasting consequences for the experience of Caribbean people – artists not least among them – who had chosen to live and work in the “mother country.”

A more nuanced understanding of this moment in the history of art would no doubt be reached through considering British nationalism as a register for issues of gender, class and age or generation. It could be deepened especially by holding up for scrutiny the Caribbean and its diaspora. When introducing the Caribbean and its transnational community to an Atlantic story of postwar art, it is less a period attitude to “race” and ethnicity that stands out, than the spatiality of its discourses and this period's Americocentrism.

It is fruitful, then, to review what was proposed for counteracting Britain's confusion about an empire and a country in “decline” relative to America. By adopting a rather Kublerian language, the phenomenon of pop art exploited the intersections of spatial and temporal distance in order to declare and render accounts of its “originality,” and to identify its “originators,” from a position outside the American-dominated story of modernism. It comprised a tale of discontinuity from a centralised high modernism which was yet continuous with narratives of British “high achievement.”

The problem for a Caribbean-born artist such as Bowling, in struggling to be remembered as integral to British pop art, is not that he was never associated with the status of the outsider or backward provincial. Quite the contrary; this was an individual who was continually reminded of his birthplace in the West Indies, of his African descent, and of “not belonging” in Britain. Bowling's predicament was rather more that he could not boast his own provincialism in the same nativist, national terms as other artists in London, nor could he contend with the evident “re-racializing” of Britain which was taking place then as a consequence of decolonization. The country's loss of empire, and those “children of empire” who migrated and “came home to roost,” were part and parcel of a nation losing out to America on the field of global dominance. Vigorous assertions of the Britishness of cultural spaces such as the field of fine art were in tension with the presence there of creative individuals from colonies and newly independent nations in the Caribbean. It was a tension that would take its toll on those artists, as we shall see, in a way that drew attention once more to the United States. Without doubt it has held lasting implications for understanding art of the Caribbean and its relation to America.

## MIGRATING TO AMERICA: CARIBBEAN ARTISTS IN MOVEMENT

It should seem unsurprising that since artists such as Bowling were given no place to belong in the British art scene of the 1960s, they should choose migration once more. As I have suggested, they were effectively left with nowhere to go but the United States. In the autumn of 1966, Frank Bowling moved to New York, marking a firm new beginning, launching the career in abstract painting for which he is now better known. Until early 1967, he lived in the Chelsea Hotel, thrilled to be sharing daily life with Jasper Johns, who also lived in the hotel, and with Andy Warhol, Rothko and many others.

The success that followed from the artist's migration is further testament to an art landscape dominated by America. Much has been made of the ways that Bowling became associated with prominent African American artists in New York, as though an identification and community through diaspora would provide the key to that success.^20^ Even so, there is a great deal in this artist's biography of movement to suggest that the diasporic concept is actually rather problematic as a tool for grasping the significance of his transatlantic experience. Claiming blackness as the basis for a countermodernity, as in much writing on the “black Atlantic,” or restating the centrality of it for an artist such as Bowling, has subjected individuals of the Caribbean to an analytical frame from which there is little to gain. This is partly due to the peculiar ways in which postwar British art had posited its “originality” in the face of “decline” and “outsiderness” relative to the United States, contending with its “lead” in the field of artistic modernism. In that UK context, ideas of displacement – which may have been thought uniquely located in postcolonial, exilic and diasporic experience – were themselves taken up in the recuperations of provincialism and temporal alterity that energized Britain's art mainstream during decolonization.

But the choice to emphasize the diasporic dimension of Caribbean experience presents a larger difficulty. It is that the prevailing conceptualization of African diaspora culture has been founded on the historical experience of the United States, rather than on a wider base of multiple locations such as those in which Caribbean artists have lived, worked and moved through. This mismatch is not inconsiderable; it is telling of what happens when discourses of cultural, racial and ethnic difference that were formed in the United States are overlain on other parts of the Atlantic. What emerged as a counterhegemonic category, in the historical struggle of people of African descent in the United States, cannot so easily be transmitted to other settings; the overall orientation toward America was experienced differently around the Atlantic. The generation of meaning and value for art and artists of the Caribbean and Britain took place in uneven ways relative to American hegemony, and was beset by competition, collisions, frictions and prejudices that mediated ideas about the United States and its perceived centrality and leadership.

The two distinctions that I began by outlining have shared this sort of paradoxical relationship. Through the effort to find a stable alternative to a displacing, centralized, developmental, modern art story, very often the same terms hauntingly return. There is the added danger of misrecognizing the same tendencies as an alternative.

There are several theorists of time and space whose ideas bear a family resemblance to Kublerian ones, with their vision of sequence and originality for the development of art at the level of its formal qualities. It is worth, for instance, seeing Paul Gilroy's idea of the black Atlantic as party to a similar scheme. With its theory of a “changing same” for black cultures in their diaspora, it isolates a developing, stylistic constant which is generated through and not despite difference and displacement. Of course, Gilroy provides a sociological account instead of an art-historical one, and he locates creativity in the outer-national or transnational experience of the descendants of enslaved Africans – those communities that are left outside nation states; indeed whose experience reveals how the formation of nationhood through modernity has commonly been a process of displacement. Yet its impact on art history has been profound, along the channel of academic practice provided by the contributions to African American studies such as that of Yale-based scholar Robert Farris Thompson, who originally coined the phrase “Afro-modern.”^21^

Between Kubler and Gilroy there is, of course, so much more to see. The decolonization movement and cultural nationalism of the 1960s, especially in the Atlantic world, in Africa and the Caribbean, yearned in a similar way for qualified and dignified difference from an “outsider” position. It pointed to ways of making transparent the sins and omissions of the past: whether that be European exploitation through centralized techno-bureaucracies, or the debasing of African ways of life and the manipulation of culture as a field of exclusions, forgetting and trauma. Even so, these histories also contribute to the normative spatio-temporality that I have outlined, with a nexus of values that may be traced to American epistemological dominance in art history.

A further example of an artist who becomes implicated in this process is Aubrey Williams (1926–90), who was also born in British Guiana. He has been the focus for much righting of wrongs in the story of British figurative and abstract painting.^22^ We are often reminded that his paintings were categorized during the 1950s and 1960s in terms of “connections” to either a “Caribbean” or a “European” heritage. Critics wrote about the “primitive urgency,” evidenced in the “tropical forests and primeval ritual dances” that they saw in his canvases.^23^ This, of course, was a familiar response to the art of artists of the Caribbean, Asia and Africa, who converged on Britain with a view to establishing themselves there during the period of the end of empire. But the description of Williams and his peers as primitive and therefore not quite modern, I would suggest, cannot be undone simply through protest, alarm, guilt, refutation and consternation, nor in some lively, emotionally powerful celebration of these artists as “truly modern,” as “countermodern,” as “black Atlantic” or even “Afro-modern.” Time and again, such gestures have invariably left in place the lingering sense of these artists as nonetheless anachronistic, and as secondary or complementary to a leading, mainstream art story. They help to rehearse an unquestioned time–space logic.

It is a logic which is not confined to a somehow inevitable exclusionary dynamic, thought to be “built in” to the canon of modernism. In decolonizing Guyana, painting itself was made anachronistic through the rejection of the medium in preference for sculpture (which was often monumental and commemorative), and public spectacle, such as in carnival, dance and other time-based, contingent media. Painting would undergo a similar fate in postwar Europe and America, although for different reasons, and through the proliferation of different sorts of media and visual technologies. This is all part of an untold story of how Aubrey Williams straddled each location, as he and his art moved between Britain, the Caribbean and, latterly, the United States. After 1966 Williams spent more and more time in America, settling finally in Florida.^24^

For an artist such as Williams it is therefore crucial to ask how he found in the conditions of provincialism and belatedness a vehicle for working through the various models of artistic practice that map onto the triangulation of the Caribbean, Britain and the United States. In this connection we may identify the challenges and limits of this artist's strivings to disabuse modernism not only of its racism but also of the politics of its geography. His success seemed to stem from an appreciation that outsiderness and anachronism have a less than essential character, and that they offer a vital margin for ambiguity, exploration, exploitation and reclamation: that the spatio-temporality of art-historical discourse may be seized upon with the aim of subversion.

Trying to express these complexities has yet to be acknowledged as a primary difficulty for studies of art and diaspora which intersect the American context during this period and are concerned with migratory lines between the Caribbean and elsewhere in its diaspora such as Britain. We need to come to grips with a Caribbean artist's aspirations to be “ahead of his time,” and a “genuine precursor.” Such an artist may have marshalled the porous simultaneity of artistic modes that have commonly been accorded a chronology (in which abstraction follows figuration), against the background of decolonization and other patterns of postwar globalizing change. The intimate details of their artworks provide the basis for an assessment of their artists' agency when set against this history and geography. Through their art, the generation of Bowling and Williams would demonstrate landmark success in turning around the accusations of being out of place and out of date.^25^

## CONTEMPORARY ART OF THE GLOBAL PROVINCES

**Figure 2.**
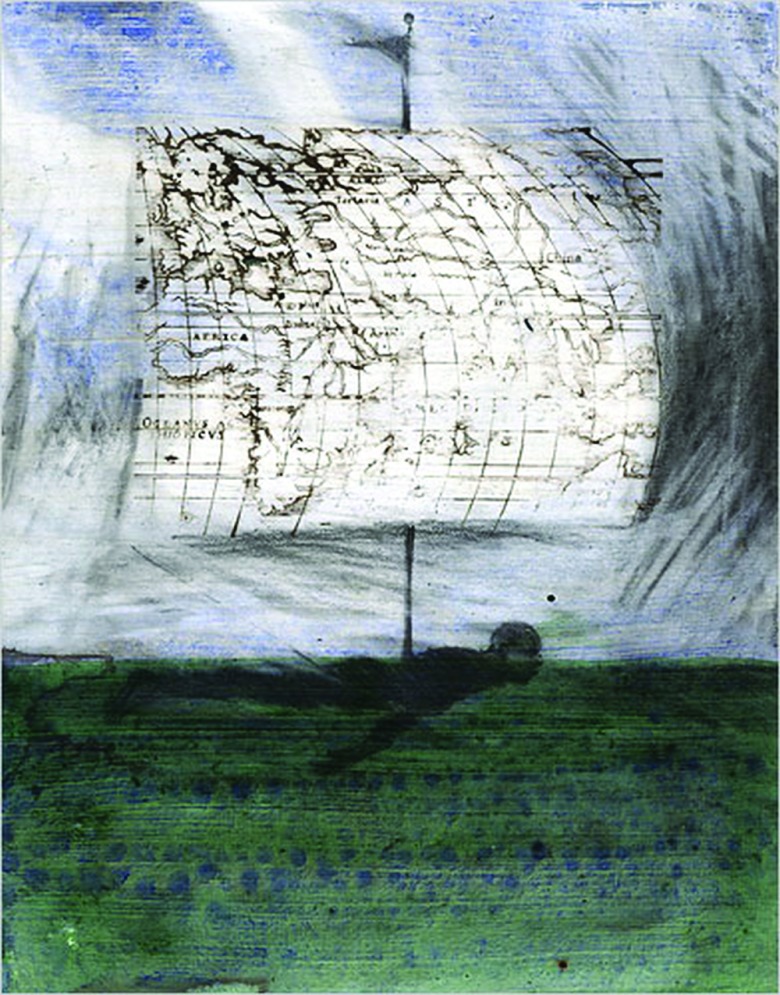
Christopher Cozier, *Castaway* (2006 and ongoing), from the Tropical Night series, ink, graphite and stamps on paper, 22·8 × 17·8 cm. Courtesy of the artist.

A notable figure on a more contemporary landscape is the artist Christopher Cozier (b. 1959), whom I have interviewed numerous times since first visiting the Caribbean in 2004. He was born in Trinidad (of the twin-island republic of Trinidad and Tobago) and returned there to live and pursue an art practice after studying undergraduate and postgraduate art degrees in the United States. Cozier's works span a range of media which includes printing, drawing, digital and video work and some performance. Figure 2 is entitled *Castaway* (2006). It features the image of a swimming figure and a map, all in an illustration style, with ink painting and pencil drawing used simultaneously. This overlapping of media comes to trouble or stretch the work's location within the category of fine art. At the same time, it shows it to be embedded in the culture of mass-produced images, circulating perhaps as illustrations to accompany printed text, or as pictorial “evidence” reproduced in a book. The composition of a body in movement presses the meaning further. Apart from connoting migration, the pictured body forms a sort of plinth or support for maps of the kind that were developed in the era of global exploration, when Europeans went on voyages around the Earth's oceans and “discovered” new territories.

Combining these details, Cozier's piece brings associations with forced movement. *Castaway* is a historical reminder that the consequences of European travel – since around the year 1400 – have included the widespread displacement of human populations, whether as the inevitable by-product of colonization or of plantation slavery. The illustrated figure is burdened by that largely untold and still unfolding story. It seems to carry a ship's sail (which is also a map) that catches the wind and is used to navigate its route. If the title of the work, *Castaway*, suggests an island location and home for the survivor of a marine disaster or misadventure, it is also the jetsam of an individual who is refused or excluded and put out of view. I take this to be Cozier's way of exploring the theme of isolation while sounding a protest at the precariousness of his own uncertain place as an artist. He is distanced from conventionally dominant “centres” of contemporary art by living “away” on a Caribbean island. This pictured figure is an active one but it also raises the issue of the choices an individual may have about whether to migrate, and what are the forms of a present-day forced migration for someone who moves in order to succeed (or simply to survive).

There are indications from *Castaway* that Cozier has actively refused the assumption that a contemporary artist is somehow “out of place” in the Caribbean. As I will suggest, this is an attitude that has prevailed historically and which artists like him have encountered on several fronts at once. The first such context is in the Caribbean itself, where certain constraints have been placed on Cozier in the course of his career. While he has always sought a “global” audience and relevance for his practice, this has often led to him being given very few opportunities in his Trinidad home.

The circumscriptions placed around the meaning and value of Cozier's art are not only externally imposed from wealthy art “centres” of the North Atlantic. These disadvantages issue from a logic of time and space which is shared as much in Trinidad as elsewhere, in which the global and local are made to appear as total opposites, through an anticolonial struggle to establish a historical periphery as a new centre. This should explain the long-standing pressure that Cozier has endured to perform the “local” role that was expected of him, as a child not of empire but of Caribbean independence, who must “serve” the Trinidad nation. Such an expectation carries the legacy of the role that was taken by nationally celebrated artists before and after the establishment of the new nation in 1962. Those artists held a quasi-official responsibility which stemmed from the ideological priority to imagine and to picture the “creolized” nation, free from colonial rule. There was an urgency to find suitable signs for an ethnically mixed and yet proudly harmonious community, fully emancipated from its past as a territory of the British Empire. What resulted was an iconography for the Caribbean that corresponded with norms and ideals of postcolonial citizenship. But it was also a scene of restrictions, working to prescribe artistic activity.

Cozier has not enjoined the battles of the anticolonial generation and he has objected to the continuing investment in its cultural politics. On completing his art education in the United States he would return to Trinidad and find a way of prompting a critical discussion of the art of the independence era. He made artworks that challenged its declarations of an absolute separation between loyal, patriotic subjects and foreign cultural “impositions” (and imposters). Over the decades that followed, Cozier has sought to decouple art in the Caribbean from such ideological priorities: the historical project of nation building and regional “federationism” – the political imagining of a community within the Caribbean's postcolonial territories. Preferring another sort of agency for art practice he has staged a thoroughgoing rejection of any “instrumentalized” role for works of art and their artists. It is a path that may have led to the sometime neglect and denigration of his art within Trinidad. In the late 1990s he would comment,

The new enemy of the nationalist has shifted from the colonizer to the perpetual “next generation” [Cozier and his peers,] whose allegedly ambiguous relationship to the national space is not understood … So the contemporary space can be interpreted as part of an ongoing evaluative or investigative look at the local, as well as the broader domain of artistic activity globally.^26^

This is an argument against the view that gaining an art education abroad is only useful if it serves domestic ends. It has shaped an art practice that shows how the old anti-imperialist counterhegemony became the new conservatism that a generation of artists have contended with since Trinidad's breaking away from Britain. The ensuing dialectic signals how the Caribbean has not wrested free from anxieties of centre and periphery in the Atlantic's cultural geography.

This first set of constraints is complicated further by Cozier's experience of operating *outside* his country and the wider Caribbean. Here he has shared challenges with other Caribbean artists which are similar to those of many more artists around the world. Like theirs, his place of birth is assumed to be a provincial, and unsuitable, zone for contemporary art. Cozier has had to cope with the supposition that the Caribbean is far too peripheral to be relevant, that it is too far removed from the centres of importance for art and its histories, and that its artists are unable to meet the particular demands of art practice at the “leading” edge. The ensuing struggle to enter the global art circuit has led the artist to pursue a record of involvements abroad that stretches back several decades. He was included recently in large group exhibitions in Liverpool and New York, and has also been curating, with several exhibitions and major art projects in Paramaribo (Suriname) and Washington, DC. The main base for his participation in exhibitions, public talks and other art events is by far the United States, and comprises a steadily growing list of activities.^27^

In contrast to the difficulties faced by the generation of Bowling and Williams – whose association with the United States seemed to hold no adverse consequences for their status as artists in the Caribbean region itself – the more recent career of an artist such as Christopher Cozier tests the paradigm of redemptive rootlessness that has been classically imputed to such diasporic biographies. The matter of where an artist may find “home” and of where a given art practice “belongs” undergoes changes as the history of art in the Caribbean unfolds. Cozier's experience differs from that of artists of previous generations such as Bowling and Williams not simply because they left the Caribbean altogether, but in the different paths that have been taken in trying to recuperate from a condition of displacement. In the current climate the demands for Caribbean “difference” are more established in the art museums and markets of the northern metropole, elevated almost to a point of principle for public arts programmers – with every risk of treating the “inclusion” of Caribbean artists as an exotic spectacle or a political commodity in a time of multiculturalism for the arts. For Cozier, the main challenge is consequently about retaining the right to choose when and where one may travel for work while maintaining a Caribbean base.

The United States dimension of Cozier's career is significant for any attempt to understand how he has coped with mobility and what has come to count as success for him and his peers. He finds himself at his most visible when participating in US exhibitions, many of which he has led as a curator, or when he undertakes an academic residency, as he did in Dartmouth College in 2007. Even so, he feels ambivalently about this, and for reasons which are quite distinct from the traditional discouragement within Trinidad to cut a figure abroad.

Such ambivalence can be elucidated by noting how works such as *Castaway* have been presented by art institutions. That work was one of several images by Cozier that make up the Tropical Night series, which was entered in the exhibition Afro Modern: Journeys through the Black Atlantic, at the UK's Tate Liverpool in 2010.^28^ The exhibition placed an emphasis on the ethnicity and “race” of artists in order to present a story of art that stretches beyond national boundaries. Cozier is of African descent, as indeed is around a third of the population of Trinidad, but within the Caribbean he has never had to present himself or his art in that connection. The significance of Cozier's participation in Afro Modern is therefore quite layered. It appears to compromise his stated interest in avoiding an ethnic or racial identification for his practice, given that the rationale for the exhibition rested on an idea of a shared identity or global community of “Afro” or “black” people. On this point it is crucial to remain in view of the Americocentrism that has underscored such art-historical and curatorial schemes for presenting and analysing art and artists of the African diaspora – in such a way as to make their “global,” transnational and transatlantic claims seem unstable.

Cozier's willingness to be included in the Afro Modern exhibition was in keeping with the artist's desire (as he put it) to become part of “the broader domain of artistic activity globally.” Even so, Afro Modern was illustrative of the conflicting purposes and meanings that can cluster around an expressly “global” exhibition. Cozier's artistic ambition is at odds with the orientation toward the United States that I have been outlining. At its simplest, this should discourage any conclusions about an absolute division or dichotomy between the local and the global, enabling a better sense of those movements across frontiers in which they entwine and complicate one another. The goal is to question those representations in which an artist of a specific location is woven into a mytho-poetics of universalist suppositions about an expanded geography that is apparently without borders.

Understanding the political economy of “the global” and contemporary art demands particular attention to such mythification. During the 1990s, much of the documentation surrounding Christopher Cozier and other artists of his generation in Trinidad suggested that “mainstream” attitudes in the art world toward the Caribbean had made it impossible to gain recognition abroad. Then, such arguments were redefined in the early 2000s, when Trinidad became the permanent home of the UK artists Chris Ofili, winner in 1998 of the prestigious Turner Prize, and the shortlisted winner Peter Doig.^29^ In response, art organizations, the art market, curators and so on around the world rapidly seized on Trinidad as an original and welcome setting for an artist to live and work. But it is disappointing that they would confine this privilege, of the “suitably distanced” place of resort, to already prominent art-world personalities.^30^ In this turn of events, Trinidad artists, including Cozier and his peers, began to self-organize more intensively. No longer were they focussed on developing alternatives to “nation narratives” (Cozier's phrase, which captures his sense of being in dialogue with the older generation of “culturalists,” or cultural nationalists).^31^ The goal instead became one of claiming transnational sovereignty, by forming more complex organizational structures for the promotion and circulation of capital and of art practices, and indeed for the promotion of artists themselves.

Since that time, artists of the transnational Caribbean have advanced their global networks, looking sometimes to the “global South,” to countries like India and South Africa. They have also turned on the older expectation that art practice ought to be supported by state patronage within the Caribbean and tried to find alternatives to the funding given by arts charities and government sponsors in Europe and North America. One consequence of these directions is that the axis of the global and the local has been scrutinized for its explanatory potential and with the aim of understanding how the experience of space is changing. I have singled out Christopher Cozier in this regard, but he is one of a great many artists who have thought about scale in particular and how it may be identified as an agent of art practice, in the search for meaningful ways to exploit the Caribbean's transnational networks.

US-based attempts to increase the visibility of the African diaspora have often extended abroad, and in doing so penetrated Caribbean territories such as Trinidad with a distinctly conscripting power. That pattern of influence mirrors the Caribbean's long-standing status as an American leisure resort and a convenient laboratory for US studies of culture and ethnicity.^32^ In Britain, curatorial practices elaborate the British–American “special relationship” in the field of the visual arts, especially when they draw upon the defining conditions and struggles around experiences of the African diaspora in North America. In the associated scholarship can be found attempts at “blackening Europe” by “making the African American experience primary” in European history. These seek to foreground the presence of North American ideas and practices – namely in the areas of literature, social studies, politics, film, dance and music – and to trace how they have travelled to Europe and changed some of the latter's traditional structures.^33^ This is what Paul Gilroy has noted, an “Americo-centric discourse” animated by “its extreme attachments to a reified notion of race.”^34^ It suggests that the possibility for establishing an expanded, Atlantic-wide historicization of the art of the African diaspora is threatened by a narrower geography and terminology.

Art-historical interest in Caribbean culture has been generally subsumed into the study of the black or African diaspora, an enterprise which is systematically framed by African American studies and some notably national priorities.^35^ The predominant uses of the diaspora concept, in both art-historical narratives and curatorial spaces, are therefore those that connect to United States-based realities, and consequently they are less suited to the more strictly transnational theorization needed for establishing an account of art and artists of the Caribbean. What has resulted is a bounded and hardly transformative representation of the African diaspora in the context of art and visual culture at least. Evidently, in the effort to find a definite break between diaspora and nationalism, as the anthropologist Aihwa Ong has warned, the “complicated accommodations, alliances, and creative tensions” that exist between them can be overlooked.^36^ The existing and most visible frameworks for historicizing art of the Caribbean, in which the black and African diaspora is central, have been unable to maintain the necessary separation from the national.

## CONCLUDING REMARKS

The Caribbean intellectuals who, in the 1960s and 1970s, fathomed the significance of decolonization for the region's cultural future sought to dispense with the overdetermination of cultural objects after the long political search for independence at the end of empire. But this period marked neither the beginning of absolute self-determination nor the end to the exercise of global influence through cultural politics. Such Caribbean experience may be examined alongside that of the Australian-born art commentator Terry Smith (another who happens to carry the imprint of Kubler). In his essay of 1974, published in the international magazine *Artforum* and entitled “The Provincialism Problem,” Smith argued, “New York remains the metropolitan center for the visual arts, to which artists living in the rest of America, in Holland, Germany, Brazil, England, France, Japan, Australia, etc. stand in a provincial relationship.”^37^ His writing showed that it is not only artists of the Caribbean that have suffered in being kept out of the histories of modern and contemporary art. While we saw a similar anxiety in British art in previous decades, Smith is interesting for noting that even New York artists were themselves prone to being provincialized. He asks us to consider those individuals who were forced to migrate to the city in order to establish their “success.” This would suggest the image of a “colonized mind” among participants in New York's art “world,” much as the writer Sam Selvon saw among British people who shared the values of its empire. Smith's observations of the 1970s could be arranged in dialogue with the subaltern thinker Dipesh Chakrabarty in his world history of the *longue dureé*.^38^ They expose for art history a vivid geography of power and a politics of temporality, through which decisions are adjudicated over who may be considered contemporary and who is marginal and belated relative to America's “locus.”

Such observations may seem accepted and even commonplace today, after the emergence in the 1970s of a social history of art and its institutions that contested the particular conception of modernism centred on abstract painting that came to hold sway in the United States during the twentieth century, and which explored the scope of modernity well beyond America.^39^ Indeed, railing against New York may itself seem pointlessly belated and provincial, even rather parochial. Yet it is undeniable that artists of the Caribbean and its diaspora, so many times set apart from their contemporaries in the wider Atlantic, are still circumscribed by models of centre and periphery that persist today and for which the United States holds a privileged place.

Apparently, artists of the Caribbean are still “catching up” with the history of art. A notable reviewer of the Brooklyn Museum's Imaginary Islands exhibition of 2007, which focussed on such artists and included works by Christopher Cozier, denied the Caribbean a place in “the contemporary” by claiming that it was above all “about identity.”^40^ This comes at a time when many Manhattan-based reviewers and curators have decided that the art world has arrived at a “post-identity” moment, even to the point of being “post-black.”^41^ The “post-” in question gestures to a putatively closed chapter in America's national history of art. So how widely enjoined are the attempts to unseat the sort of geography I have described for artists of the African diaspora that underscores Americocentrism? Despite all these “posts” it is certainly business as usual in art curating and criticism. We are evidently still contending with what the artist and curator Rasheed Araeen once so aptly called “the citadels of modernism,”^42^ although these are best apprehended as temporal locations as much as physical spaces. The Caribbean and its diaspora elsewhere around the shores of the Atlantic could be separated from a history of art in the United States, but that would be an error. Any such discrete narration would induce worse distortions than the time–space logic surrounding the more visible contours of knowledge about art of the Caribbean.

## Footnotes

1 David Summers, Real Spaces: World Art History and the Rise of Western Modernism (New York: Phaidon Press, 2003), 201. See also some remarks on centre–periphery relations in the history of art: Thomas DaCosta Kaufmann, Toward a Geography of Art (Chicago: The University of Chicago Press, 2004); and Piotr Piotrowski, “Between Place and Time: A Critical Geography of “New” Central Europe,” in ibid., 153–71. In general, these follow Foucault's suggestion: “The spatializing description of discursive realities gives on to the analysis of related effects of power.” Michel Foucault, Power/Knowledge: Selected Interviews and Other Writings, 1972–1977 (New York: Pantheon Books, 1980), 70–71.

2 Karl Marx, The Eighteenth Brumaire of Louis Bonaparte (Moscow: Progress Publishers, 1937; first published 1852).

3 Johannes Fabian, Time and the Other: How Anthropology Makes Its Object (New York: Columbia University Press, 1983), 16.

4 George Kubler, The Shape of Time: Remarks on the History of Things (New Haven and London: Yale University Press, 1962), 91.

5 Kubler, 114.

6 Morris's master's thesis approached Brancusi through Kubler's terms. See A. Reinhardt, “Art vs. history,” Art News, 64, 19 (1966), 19–21; cf. W. Smith, “Ad Reinhardt's oriental aesthetic,” Smithsonian Studies in American Art, 4, 3–4 (1990), 22–45. Smithson's connections with Kubler can be seen in J. Flam, ed., Robert Smithson: The Collected Writings (Berkeley: University of California Press, 1996). A detailed examination of Kubler's influence on Smithson is given in Pamela M. Lee, “ ‘Ultramoderne’: Or, How George Kubler Stole the Time in Sixties Art,” Grey Room, 2 (2001), 46–77; Lee, Chronophobia: On Time in the Art of the 1960s (Cambridge, MA: MIT Press, 2004).

7 David Summers, Real Spaces: World Art History and the Rise of Western Modernism (London: Phaidon Press, 2003), 16.

8 D. Robbins, The Independent Group: Postwar Britain and the Aesthetics of Plenty (Cambridge, MA: MIT Press, 1990).

9 David E. Brauer, Jim Edwards, Christopher Finch, Walter Hopps and Ned Rifkin, eds., *Pop Art: US/UK Connections, 1956–1966* (Houston, TX: Menil Collection and Hatje Cantz Publishers, 2001), 17.

10 P. Fuller, “The Visual Arts,” in B. Ford, ed., Modern Britain: The Cambridge Cultural History, Volume IX (Cambridge: Cambridge University Press, 1992), 99–145, 99.

11 Len Deighton, “Foreword,” in Alex Seago, Burning the Box of Beautiful Things: The Development of a Postmodern Sensibility (Oxford: Oxford University Press, 1995), vii–xii, xi–xii.

12 Middleton is quoted in Peter Fuller, “The Neo-Romantics: London, Barbican Art Gallery,” Burlington Magazine, 129, 1012 (1987), 472–74, 474.

13 R. B. Kitaj, The Human Clay: An Exhibition (London: Arts Council of Great Britain, 1976).

14 S. Compton, ed., British Art in the Twentieth Century: The Modern Movement (London: Royal Academy of Arts, 1987).

15 R. Shone and P. Fuller, “British Art in the Twentieth Century: London, Royal Academy,” Burlington Magazine, 129, 1009 (1987), 261–65, 265.

16 From among the literature which is contemporary with pop art see A. Bowness, “The American Invasion and the British Response,” Studio International, 173, 890 (1967), 285–93; L. R. Lippard, ed., *Pop Art* (London: Thames and Hudson, 1966); S. Gablik and J. Russell, Pop Art Redefined (London: Thames and Hudson, 1969).

17 There is an even wider literature here, but key examples include Charles Harrison, “ Modernism and the ‘Transatlantic Dialogue’,” in F. Frascina, ed., Pollock and After: The Critical Debate (London: Harper and Row, 1985), 217–32; Marco Livingstone, ed., Pop Art (London: Royal Academy of Arts and Weidenfield and Nicholson, 1991); J. A. Walker, Cultural Offensive: America's Impact on British Art since 1945 (London: Pluto, 1998); Nigel Whiteley, “‘Cultural Imperialism’? British Hard-Edge Painting in the 1960s”, Third Text, 22, 2 (2008), 209–27.”

18 Quoted in Greil Marcus, “No Money Down: Pop Art, Pop Music, Pop Culture,” in M. Francis and H. Foster, eds., Pop: Themes and Movements (London: Phaidon, 2005), 208–10, 208. See also L. Alloway, “Avant-Garde, London,” Image, Oct. 1960, 40.

19 Anne Walmsley, The Caribbean Artists Movement: 1966–1972 (London: New Beacon Books, 1992).

20 Kobena Mercer, “Black Atlantic Abstraction: Aubrey Williams and Frank Bowling,” in Mercer, ed., Discrepant Abstraction (London and Cambridge MA: Institute of International Visual Arts and MIT Press, 2006), 184–85.

21 Robert Farris Thompson, “Afro-Modernism,” Artforum, 30 (Sept. 1991), 91–94.

22 Walmsley; Walmsley, ed., Guyana Dreaming: The Art of Aubrey Williams (Aarhus: Dangeroo Press, 1990); Wilson Harris, “Aubrey Williams,” Third Text, 10, 34 (1996), 79–82; Mercer, 184–85.

23 Eric Newton, “Round the London Galleries,” The Listener, 28 Aug. 1958, 310. This passage is quoted, but incorrectly cited, in Walmsley, *The Caribbean Artists Movement*, 17.

24 See Anne Walmsley, “Chronology,” in Andrew Dempsey, Gilane Tawadros and Maridowa Williams, eds., Aubrey Williams (London: Institute of International Visual Arts, 1998), 65–101.

25 Leon Wainwright, Timed Out: Art and the Transnational Caribbean (Manchester: Manchester University Press, 2011).

26 Christopher Cozier, “Between Narratives and Other Spaces,” Small Axe, 6 (Sept. 1999), 19–37, 22.

27 *The Global Africa Project* (Museum of Arts and Design and the Center for Race and Culture, Maryland Institute College of Art, 2010). With Tatiana Flores, *Cozier was cocurator of* Wrestling with the Image: Caribbean Interventions (Washington, DC: World Bank Art Program and Art Museum of the Americas, Organisation of American States, 2011). An accompanying exhibition catalogue is published online at www.artzpub.com/sites/default/files/pdf/wwtil.pdf. Other recent art projects in the US include: SSV4KY, a collaborative sound installation (University of Kentucky Department of Art and the Kentucky Museum of Art and Craft, 2012), and Into the Mix (Kentucky Museum of Art and Craft, 2012).

28 Tanya Barson and Peter Gorschlüter, eds., Afro Modern: Journeys through the Black Atlantic (exhibition catalogue) (Liverpool and London: Tate, 2010). See also www.tate.org.uk/liverpool/exhibitions/afromodernism/default.shtm.

29 These artists are represented by leading art dealers in London and New York and in Germany, and are known to be among the top five wealthiest living artists from Europe. In 2007, a resale of Peter Doig's painting *White Canoe* at Sotheby's auction house fetched $11.3 million. Chris Ofili's *The Upper Room* was purchased in 2005 by Tate for £600,000 and installed at Tate Britain, in collaboration with the UK architect David Adjaye.

30 Judith Nesbitt, “A Suitable Distance,” in Nesbitt, ed., Peter Doig (London: Tate Publishing, 2008), 9–20; Nesbitt, ed., Chris Ofili (London: Tate Publishing, 2010). An exhibition in Trinidad in 2006 attempted to bridge the gap I have described by grouping works by Ofili and Doig together with those by other expatriate artists, several of whom had moved to Trinidad from elsewhere in the Caribbean. See Leon Wainwright, “Apples and Grapes ‘from Foreign’: Five Figures in a Landscape,” in Andy Jacob, ed., A Suitable Distance: Rex Dixon, Peter Doig, Kofi Kayiga, Chris Ofili and Roberta Stoddard. (Trinidad: Soft Box Studios, 2006), 4–11.

31 See http://christophercozier.blogspot.com. This was suggested by Cozier's installation and performance work Conversation with a Shirt-jac (1991). See Annie Paul, “The Enigma of Survival: Travelling beyond the Expat Gaze,” Art Journal, 62, 1 (2003), 48–67.

32 For a history of this involvement see the final chapter in Mimi Sheller, Consuming the Caribbean: From Arawaks to Zombies (London: Routledge, 2004). An extended bibliography appears in Aisha Khan, Callaloo Nation: Metaphors of Race and Religious Identity among South Asians in Trinidad (London and Durham, NC: Duke University Press, 2004).

33 See, for example, Heike Raphael-Hernandez, ed., Blackening Europe: The African American Presence (London and New York: Routledge), 1–12.

34 Paul Gilroy, “Foreword,” in ibid., xvi. Gilroy's contribution to this volume is all the more fascinating for its cogent critique of the overall approach of the book. It develops on his earlier observations about nationalist tendencies among black American intellectuals. Paul Gilroy, The Black Atlantic: Modernity and Double Consciousness (Cambridge, MA: Harvard University Press, 1992).

35 See, for example, Gina Dent, ed., Black Popular Culture: A Project by Michele Wallace (Seattle: Bay Press, 1992); M. Harris, Colored Pictures: Race and Visual Representation (Chapel Hill: University of North Carolina Press, 2003); P. Farris-Dufrene, ed., Voices of Color: Art and Society in the Americas (Atlanta Highlands: Humanities Press, 1997); bell hooks, Art on My Mind: Visual Politics (New York: The New Press, 1995); cf. Petrine Archer-Straw, David A. Bailey and Richard Powell, eds., Back to Black: Art, Cinema and the Racial Imaginary (London: The Whitechapel Gallery, 2005); Sally Price and Richard Price, Romare Bearden: The Caribbean Dimension (Philadelphia: University of Pennsylvania Press, 2006).

36 Aihwa Ong, Flexible Citizenship: The Cultural Logics of Transnationality (Durham, NC and London: Duke University Press, 1999), 16.

37 Terry Smith, “The Provincialism Problem,” Artforum, 13 (1974), 54–59, 56.

38 Dipesh Chakrabarty, Provincializing Europe: Postcolonial Thought and Historical Difference (Princeton: Princeton University Press, 2000).

39 Francis Frascina and Charles Harrison, eds., Modern Art and Modernism: A Critical Anthology (New York: Harper and Row, 1982); Victor Burgin, “The End of Art Theory,” in Burgin, The End of Art Theory: Criticism and Postmodernity (London: Palgrave Macmillan, 1986), 140–215; Craig Owens, “Representation, Appropriation, and Power,” in Owens, Beyond Recognition: Representation, Power, and Culture (Berkeley: University of California Press, 1992), 88–113; T. J. Clark, Farewell to an Idea: Episodes from a History of Modernism (New Haven and London: Yale University Press, 1999).

40 Holland Cotter, “Caribbean Visions of Tropical Paradise and Protest,” New York Times, 31 Aug. 2007.

41 Thelma Golden, Freestyle, exhibition catalogue (New York: Studio Museum in Harlem, 2001), 14; cf. Darby English, How to See a Work of Art in Total Darkness (Cambridge, MA: MIT Press, 2007).

42 Rasheed Araeen, ed., The Other Story: Afro-Asian Artists in Post-war Britain (London: The Hayward Gallery, 1989).

